# Interdisciplinary approach in emergency revascularization and treatment for acute mesenteric ischemia

**DOI:** 10.1186/s12893-021-01102-9

**Published:** 2021-02-18

**Authors:** Alicja Zientara, Anja-Rebeka Domenghino, Igor Schwegler, Hans Bruijnen, Annelies Schnider, Markus Weber, Stefan Gutknecht, Nicolas Attigah

**Affiliations:** 1 Department of Cardiothoracic Surgery, Royal Brompton and Harefield Hospital, Harefield, UK; 2 Department of Vascular Surgery, Triemli Hospital, 497, 8063 Zurich, Switzerland; 3 Augsburg City Hospital, Augsburg, Germany; 4 Department of Visceral Surgery, Triemli Hospital, Zürich, Switzerland

**Keywords:** Acute mesenteric ischemia, Bowel resection, Intestinal ischemia, Mesenteric revascularization, Iliac-mesenteric bypass, Thrombembolectomy of superior mesenteric artery

## Abstract

**Background:**

Mesenteric ischemia is associated with poor outcome and high overall mortality. The aim was to analyze an interdisciplinary treatment approach of vascular and visceral specialists focusing on the in-hospital outcome and follow-up in patients with acute and acute-on-chronic mesenteric ischemia.

**Methods:**

From 2010 until 2017, 26 consecutive patients with acute or acute on chronic mesenteric ischemia were treated by an interdisciplinary team. Data were prospectively collected and retrospectively evaluated. Throughout the initial examination, the extent of bowel resection was determined by the visceral surgeon and the appropriate mode of revascularization by the vascular surgeon. The routine follow-up included clinical examination and ultrasound- or CT-imaging for patency assessment and overall survival as primary endpoint of the study.

**Results:**

Out of 26 patients, 18 (69.2%) were rendered for open repair. Ten patients (38.5%) received reconstruction of the superior mesenteric artery with an iliac-mesenteric bypass. Seven patients (26.9%) underwent thrombembolectomy of the mesenteric artery. One patient received an infra-diaphragmatic aorto-celiac-mesenteric bypass. Out of the 8 patients, who were not suitable for open revascularization, 2 patients (7.7%) were treated endovascularly and 6 (23.1%) underwent explorative laparotomy.

The in-hospital mortality was 23% (n = 6). The mean survival of the revascularized group (n = 20) was 51.8 months (95% CI 39.1–64.5) compared to 15.7 months in the non-revascularized group (n = 6) (95% CI − 4.8–36.1; p = 0.08). The median follow-up was 64.6 months. Primary patency in the 16 patients after open and 2 after interventional revascularization was 100% and 89.9% in the follow-up.

**Conclusion:**

The interdisciplinary treatment of mesenteric ischemia improves survival if carried out in time. Hereby open revascularization measures are advantageous as they allow bowel assessment, resection, and revascularization in a one-stop fashion especially in advanced cases.

## Introduction

Despite various open and endovascular treatment approaches acute and acute-on-chronic mesenteric ischemia is still associated with a poor outcome with mortality rates between 60 and 80% in an acute setting [1–3]. The most common cause of mesenteric ischemia is arterial embolization or arterial thrombosis of the superior mesenteric artery (SMA), but it can also be a highly lethal complication in aortic dissection [4].

Early diagnosis is the crucial parameter in treating acute mesenteric ischemia (AMI) successfully. However, the clinical presentation is often inconsistent and laboratory testing frequently unreliable. As signs of peritonitis are often absent, even though disproportional pain is reported, the typically transient amelioration of symptoms is deceptive and may lead to an irrecoverable treatment delay. Even if an appropriate diagnosis is established in time it usually takes an interdisciplinary team approach including vascular, visceral surgery, and interventional radiology for treatment.

As European guidelines favor endovascular treatment in a subset of clinical settings the role of the appropriate therapeutic modality in AMI remains controversial [5–7]. The aim of this study is to analyze the outcome of an interdisciplinary team approach to treat acute mesenteric ischemia.

## Methods

Between April 2010 and July 2017, 26 consecutive patients in a single center underwent treatment for acute or acute-on-chronic mesenteric ischemia. Data were collected prospectively in a database and retrospectively investigated. After discharge, patients were included in a routine follow-up regimen with clinical, sonographic, and radiologic evaluation by Angio-CT scans 6 weeks and 3 months postoperatively followed by yearly scans. Primary endpoints of the study were long-term overall survival and primary patency of the vascular reconstruction. Patients gave informed consent to the operation and the encrypted use of clinical data for research purposes.

### Diagnostic and treatment algorithm

Patients with clinical findings or medical history suggestive for mesenteric ischemia received a primary clinical survey by a visceral and a vascular surgeon. Concomitant laboratory testing including white blood count, electrolytes and serum lactate was obtained. To confirm suspected bowel ischemia the most sensitive imaging modality is a 3-phase Angio-CT [5]. The CT localizes the type of occlusion (i.e. thrombosis, embolism, dissection), the affected vessels and gives an estimate of the intestinal damage to be expected. As soon as the diagnosis was confirmed, emergency exploratory laparotomy was performed. Intraoperatively the decision concerning the necessity of bowel resection and type of revascularization was made taking the preoperative imaging into account.

In cases of evidentially short onset of symptoms with neither clinical nor radiological signs of advanced bowel ischemia, endovascular revascularization was considered as primary treatment option with close post-interventional surveillance.

Endovascular Technique:

Usually transfemoral access was obtained for angiography of the visceral aorta. Alternatively, left brachial access was chosen to increase pushability and optimized angle for recanalization of mesenteric artery or celiac trunk occlusion or in cases of extensive atherosclerotic disease of the aorta and iliac vessels.

## Surgical technique

### Iliac-mesenteric bypass

**Fig. 1:**
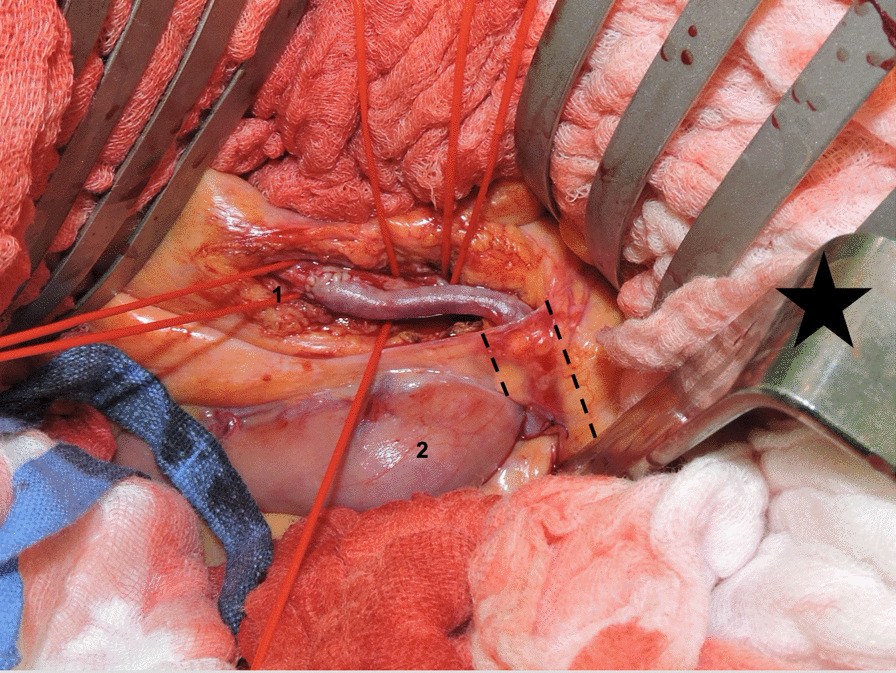
Intraoperative view of an aorto-mesenteric bypass with a reversed saphenous vein graft: 1: end-to-side anastomosis to the proximal mesenteric artery (SMA), 2: proximal jejunum; dotted lines: Ligament of Treitz with tunneled bypass. x, *: cephalad direction

The cornerstone of emergency mesenteric revascularization is the iliac-mesenteric bypass via a median laparotomy. With this extra-anatomic approach also challenging types of occlusion, like in aortic dissection, can be managed. Usually, the left or right common iliac artery was exposed, alternatively, according to the degree of calcifications, the external iliac arteries were used. The SMA was approached ventrally by holding the transverse colon cephalad. It is crucial to place the bypass retroperitoneal in order to create a stable course. Therefore, the retroperitoneum covering the aorta had to be opened from the iliac artery to the left renal vein. Afterwards, the bypass was covered with retroperitoneal tissue by a suture closing the peritoneum. To avoid kinking the bypass, it should be laid in a “lazy C”-manner using the ligament of Treitz as a pivot (Fig. 1).

### Infra-diaphragmatic aorto-celiac-mesenteric bypass

A bilateral-subcostal incision was performed. After mobilization of the left liver lobe and dissection of the diaphragmatic crus, the supra-celiac aorta was exposed through the hepato-gastric ligament. As described above, the SMA was exposed anteriorly. We preferred an end-to-side fashion to perform the proximal and distal anastomoses. Usually, the mesenteric branch can be advanced downwards in a retro-pancreatic position. This approach is far more elaborate and only suitable in cases of stable patients without gut necrosis or signs of intestinal bacterial translocation.

### Thrombembolectomy of the SMA

The SMA was exposed the same way as described above. In the case of sufficient vessel diameter, the Fogarty-maneuver can be carried out over a transversal arteriotomy. In case of very small or calcified vessels, a longitudinal incision with patch-closure can be useful. Dissection should include the proximal branches of the SMA which can be embolectomized selectively.

### Technique of bowel resection

A minimum length of 150 cm of small bowel was judged as necessary for survival. Small bowel was resected to the lowest extent as possible. In the presence of areas in doubt of potential recovery a second look laparotomy was indicated with a low threshold. By no means primary anastomoses were carried out, in case of bowel discontinuity stoma with delayed anastomosis was preferred.

### Statistical analysis

Descriptive statistics were generated by calculation the percentages of nominal and ordinal variables. Numeric variables were described with median and interquartile range (IQR). Survival analysis was done by the method described by Kaplan and Meier. Differences between the groups were calculated with the log-rank test. Statsdirect software (Version 2.7.3, Statsdirect Ltd, Cheshire, UK) was used for all statistical analyses.

## Results

### Patient characteristics and findings on computed tomography

Seventeen patients (65.4%) presented with acute and 9 (34.6%) with acute-on-chronic mesenteric ischemia. Patients' characteristics, CT findings and laboratory values are summarized in Table 1. Ten patients (38.5%) had specific (pneumatosis intestinalis, portal gas) or non-specific signs (free gas or fluid) for AMI on the CT scan, whereas 16 (61.5%) patients did not show any signs for AMI in the imaging.

**Table 1:** Patient characteristics and CT findings

Parameter	Patients (n = 26)
Age: y (median)	75 (IQR 64–99)
Female	12 (46.1%)
ASA:	
1	0%
2	4 (15.4%)
3	18 (69.2%)
4	1 (3.9%)
5	3 (11.6%)
Comorbidity:	
Diabetes	9 (34.6%)
Arterial Hypertension	22 (84.6%)
Dyslipidemia	9 (34.6%)
COPD	10 (38.5%)
PAD	13 (50%)
CAD	9 (34.6%)
Biochemical data:	
White blood cell count^a^	12.95 (IQR 9.6–17.7)
Lactate^a^	2.85 (IQR 1.4–6.4)
CT-findings^b^:	(38.5%)
Intestinal pneumatosis	4
Gas in portal vein	3
Paralytic ileus	2
Free fluid	2
Free gas	1
Wall thickening	7

*ASA* American Society of Anesthesiologists, *COPD* chronic obstructive pulmonary disease, *PAD* peripheral arterial disease, *CAD* coronary arterial disease, *IQR* interquartile range

^a^White blood cell count and lactate serum levels at initial diagnosis (normal values: leucocytes 3.6–10.5 × 10^9^/l; lactate 0.5–2.2 mmol/l)

^b^CT-findings at initial diagnosis (including 4 missing values), tabulated as rather specific (intestinal pneumatosis and gas in the portal vein system) and non-specific findings (free gas/fluid, etc.)

### Patients with revascularization

**Fig. 2:**
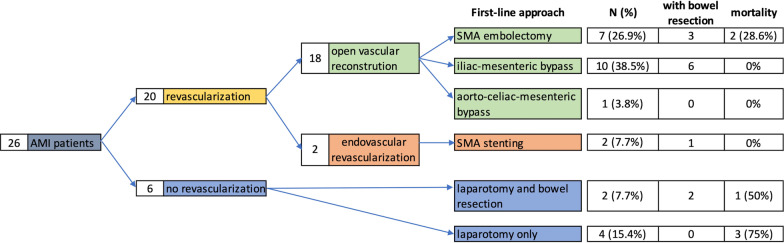
Overview of the total number of patients and revascularization technique

The detailed procedures with and without concomitant bowel resection and in-hospital mortality are demonstrated in Fig. 2. Out of 26 patients, 18 (69.2%) received an open vascular reconstruction, 2 patients an endovascular revascularization, and in 6 patients no revascularization was performed.

The majority of the patients treated with open revascularization had a vascular reconstruction of the SMA with an iliac-mesenteric bypass (n = 10, 38.5%). In 7 patients a saphenous vein graft, in 1 patient the basilic vein, and in 2 patients a PTFE graft was used.

Seven (26.9%) patients underwent embolectomy of the mesenteric artery with a Fogarty-maneuver. One patient (3.8%) received an infra-diaphragmatic aorto-celiac-mesenteric Dacron bypass (Gelsoft 14 × 7 mm) for a two-vessel disease. 9 out of 18 patients with open reconstruction underwent concomitant small or large bowel resection.

Two patients (7.7%) received stenting of the SMA. In the endovascular group, 1 patient underwent an emergency laparotomy including small bowel resection and open-abdomen treatment 5 days before the endovascular intervention.

### Patients without revascularization

Out of 6 patients where revascularization was judged as not feasible due to very distal embolism or did not seem promising due to extensive lethal bowel ischemia, two patients survived throughout the long-term follow-up after undergoing emergent bowel resection. In both cases the infarcted area was localized peripherally and affected single intestinal loops which could be managed successfully by simple resection (Fig. 2). In the remaining four patients with progressed mesenteric ischemia the abdomen was closed and palliative treatment was established in the presence of beginning multiorgan failure and sepsis.

### Complications and second look

Sixteen out of 26 (61.5%) patients had complications during the postoperative course. Two patients (7.7%) underwent reoperation for bleeding and hematoma after vascular reconstruction and 3 patients (11.5%) experienced cardiac complications through myocardial infarction (STEMI, NSTEMI). Eight patients (30.8%) were treated with intermittent hemodialysis due to renal failure. The remaining adverse events were minor, consisting mainly of wound complications.

Six patients (23.1%) received a planned second-look operation. Whilst 3 patients underwent bowel resection, the other 3 patients had a secondary closure of the abdomen without additional intervention.

### In-hospital mortality and survival

Six of the 26 patients (2/18 after open reconstruction, 4/8 without revascularization) died in hospital (23.1%) due to the large extent of the mesenteric ischemia and septic condition. Apart from one patient, who died on day 6 postoperatively, all deaths occurred within the first 24 h after laparotomy.

**Fig. 3:**
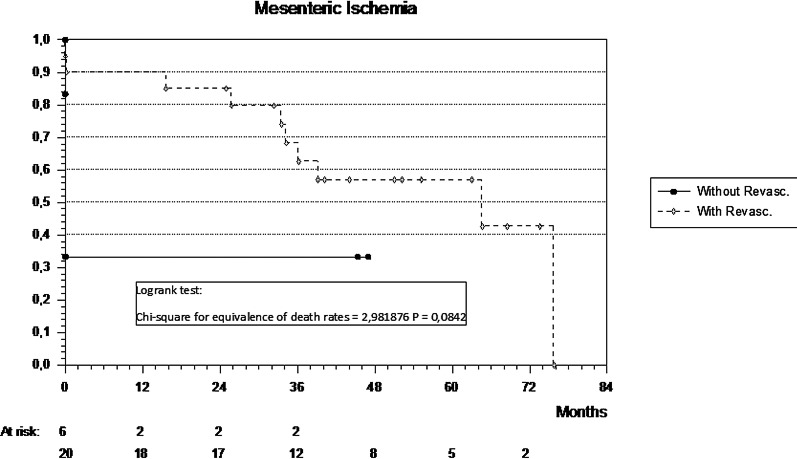
Kaplan–Meier survival curve of the revascularized patients (n = 20) vs no revascularization (n = 6)

The median follow-up of the cohort was 64.6 months. The mean survival of the revascularized group of 20 patients was 51.8 months (95% CI 39.1–64.5) compared to shorter mean survival of 15.7 months in the non-revascularized group (n = 6) (95% CI -4.8–36.1; p = 0.08 [calculated power of 0.76]) (Fig. 3).

### Patency

Primary patency at discharge in the 16 patients after open and 2 after interventional revascularization was 100%. The overall bypass patency in the time frame of the clinical follow-up was 89.9% accounting for 2 patients who developed failure of the revascularization. One of the patients with SMA stenting had to be re-operated due to recurrent in-stent stenosis with an infra-diaphragmatic aorto-mesenteric-hepatic Y-bypass. The reoperation was in the time frame of 5 years and the patient presented with a good short-term follow-up 90 days after the second operation. Another patient after open embolectomy and aorto-mesenteric bypass needed reoperation for bypass failure 5 years after the initial operation with an iliac-mesenteric bypass and presented in good health 30 days after discharge in the outpatient clinic.

### Gastro-intestinal resections and clinical follow-up

In 9 of the 18 revascularized patients, bowel resection was necessary (5 × small bowel, 1 × large bowel, 3 × small and large bowel). Five patients received a stoma, which could be reversed in all patients over the following course. Two patients developed short bowel syndrome with the need for oral nutritional supplementation. The patient undergoing an emergency laparotomy with small bowel resection, open-abdomen treatment for 5 days, and thereafter endovascular intervention, presented without any intestinal symptoms at the 3-years follow-up.

Three out of the four of the patients receiving only laparotomy with bowel resection died in hospital within 24 h due to the rapid progression of ischemia.

## Discussion

Our study shows that interdisciplinarity of vascular and visceral surgeon specialists in the treatment of acute onset mesenteric ischemia is associated with improved outcome probability. In a cohort of 26 consecutive patients presenting with acute or acute on chronic mesenteric ischemia the mean survival was 51.8 months (95%CI 39.1–64.5) compared to 15.7 months in the non-vascularized group (n = 6) (95% CI4.8–36.12; p = 0.08) in a 64.6 months follow-up. The overall mortality in our cohort was 23%. This is consistent with other series. Cho et al. reported in 48 patients with AMI a perioperative mortality of 52% [2]. Data from the Swedvasc registry reported a thirty-day mortality rates from 42 and 58% for patients treated by endovascular means and open revascularization, respectively. Primary patency in our predominantly open revascularized patients was 100% in the open group and 89.9% in the endovascular patients. Cho et al. reported a cumulative patency of 57% in five years including a historic patients sample surveying a 37-year experience [2]. McMillan et al. reported a patency rate for mesenteric artery bypass grafts of 89% at 72 months [8].

Angio-CT scan has become far more sensitive and is the imaging modality of choice for AMI. Therefore, the European Society of Vascular Surgery (ESVS) recommends angio-CT as initial investigation as class I, evidence level B [5]. In addition to the detection of involved vessels or the type of occlusion (thromboembolism, atherosclerotic, or dissection) the angio-CT can also show typical sequela of prolonged intestinal ischemia like gut wall thickening, free fluid/air, intestinal pneumatosis, or gas in the portal vein system. However, it has been documented that in AOCMI (acute-on-chronic mesenteric ischemia) around one-third of patients do not show any signs of ischemia on the CT scan [9]. This is in accordance with our results where 54.6% of the patients with a diagnosed acute mesenteric ischemia did neither show any specific signs like intestinal pneumatosis or gas in the portal vein system, nor non-specific findings like free gas or fluid in the abdominal cavity. In this acute scenario, initial laboratory testing was not supportive in our results with huge variation in white blood cell count and serum lactate levels on admission [10, 11].

Thus, our threshold for explorative laparotomy was low and only patients with a short onset of symptoms and without the abovementioned findings on CT scan were rendered to primary endovascular approach. Out of those, who revascularization was judged as not feasible or did not seem promising, two patients survived throughout the long-term follow up, one of them after emergency bowel resection. This experience underscores the importance of the explorative laparotomy as a diagnostic and life-saving tool in the scenario of clinical symptoms without specific ischemia-related signs in the imaging.

Abdominal exploration followed by vascular bypass has been the standard of care for AMI, but there is increasing use of endovascular treatment with and without exploratory laparotomy. The use of mechanical and aspiration embolectomy, sometimes in combination with thrombolytic therapy, is often successful in offering a treatment alternative to open surgical revascularization [12]. Compared to open surgery the endovascular approach may be preferred in elderly and fragile patients and is supported by the current guidelines of the ESVS [5, 13]. Measurable advantages have been reported by Arthurs et al. showing reduced in-hospital mortality of 36% versus 50% in the surgically revascularized group with AMI. However, the majority of the patients were treated with an endovascular approach (n = 56) and only 10 patients received an open bypass surgery [14]. In a retrospective cohort with patients after endovascular intervention, Hsu et al. demonstrated that short time-to-reperfusion was significant in predicting survival for patients who underwent additional exploratory laparotomy. They concluded emergent endovascular treatment before laparotomy might be associated with a better survival [15]. Direct endovascular revascularization during laparotomy can be feasible but needs well-equipped imaging modalities and is so far not well established [16]. These results emphasize that a successful treatment of AMI might be mainly a question of timing and that despite potential endovascular means, laparotomy remains a necessary life-saving treatment.

Zettervall et al. found in a large cohort with over 14.000 patients that despite the significant growth of endovascular interventions, the frequency of embolectomy for AMI remained unchanged and the rate of open surgery for chronic and acute mesenteric ischemia remained stable over 12 years [17]. Even at centers of excellence in endovascular treatment, 88% of patients between 1999 and 2010 underwent open revascularization, without dramatic changes in open treatment over time [6].

In the open approach, the visceral surgeon can assess accurately the intestinal viability, the extent of resection if needed, probable degree of intestinal recovery, and the need for a second look operation. In patients treated initially with an endovascular approach, this crucial part of treatment is lost or at least postponed. Additionally, if the bowel ischemia has already progressed to a certain degree, purely endovascular treatment might be unsuccessful. Frequently, bowel viability after reperfusion cannot be determined with certainty at the time of initial exploration. The frequency of bowel resection is higher during second-look surgery (53%) compared to the initial exploration (31%) which underlines the importance of a two-staged open approach [18].

Another important consideration supporting the open revascularization might be the potentially longer durability and patency compared to endovascular stenting. Though difficult to prove, as there is only limited data available, few sources documented a rate of re-stenosis in the first two years after stenting in chronic ischemia of 28–55% compared to publications on open bypass surgery with re-stenosis rate of 0–25% [3, 19–21]. As shown in one of our patients after endovascular treatment, repetitive interventions were necessary to keep the stent patent, which finally led to open revascularization. However, the individual decision in this emergency case may not be representative to draw a general conclusion for standard treatment, neither interventional nor surgical.

Considering the technical aspects, the iliac-mesenteric bypass via an anterior transabdominal approach is the easiest way to re-establish mesenteric perfusion in an emergency setting if thrombembolectomy is not indicated or feasible. Some authors advocate the so-called “French bypass” where the bypass passes the left renal pedicle [22]. The advantage of this approach has been described to avoid bypass kinking and providing enough length to adapt to movements of the SMA. The proximal anastomosis is retrograde on the left side of the infrarenal aorta. The course of the bypass runs first in the back and top of the retro-renal dissection plane, then loops behind and over the left renal pedicle, and finally turns downward and forward to the SMA. In our opinion, it is sufficient to use the ligament of Treitz as a pivot to avoid kinking of the bypass and create the "lazy C shape" bypass course (Fig. 1). We use this technique routinely, which requires less retroperitoneal dissection.

However, this study is flawed by relatively small sample size, heterogeneous composition and retrospective design. In our study 12 patients (46,2%) needed bowel resection at initial assessment and seven patients (26,9%) received a second look laparotomy. This emphasizes the role of both, reconstructive vascular measures in combination with prompt intestinal assessment and resection if required, in successful treatment of intestinal malperfusion [23].

## Conclusion

Encouraging early and late survival rates can be achieved if mesenteric ischemia is diagnosed on time. The assessment and therapy should be carried out by an interdisciplinary approach keeping the time-to-reperfusion as short as possible. Open revascularization might stay the treatment of choice in case of unclear imaging and advanced ischemia. Intraperitoneal revascularization with iliac-mesenteric bypass showed good patency rates in the mid- to long-term follow-up.

## Data Availability

The datasets generated and/or analyzed during the current study are not publicly available due to patient privacy and security of electronic medical information but are (anonymized) available from the corresponding author on reasonable request.
